# Microfluidic Organ/Body-on-a-Chip Devices at the Convergence of Biology and Microengineering

**DOI:** 10.3390/s151229848

**Published:** 2015-12-10

**Authors:** Ana Rubina Perestrelo, Ana C. P. Águas, Alberto Rainer, Giancarlo Forte

**Affiliations:** 1 International Clinical Research Center (ICRC), Integrated Center of Cellular Therapy and Regenerative Medicine (ICCT), St. Anne’s University Hospital, Brno 656 91, Czech Republic; 2 Center for Biomedical Research, University of Algarve, Faro 8005-139, Portugal; acaguas@ualg.pt; 3 Tissue Engineering Unit, Università Campus Bio-Medico di Roma, Rome 00128, Italy; a.rainer@unicampus.it; 4 Department of Biomaterials Science, University of Turku, Turku 20014, Finland

**Keywords:** microfluidics, BioMEMs, organ-on-a-chip, body-on-a-chip, tissue engineering

## Abstract

Recent advances in biomedical technologies are mostly related to the convergence of biology with microengineering. For instance, microfluidic devices are now commonly found in most research centers, clinics and hospitals, contributing to more accurate studies and therapies as powerful tools for drug delivery, monitoring of specific analytes, and medical diagnostics. Most remarkably, integration of cellularized constructs within microengineered platforms has enabled the recapitulation of the physiological and pathological conditions of complex tissues and organs. The so-called “organ-on-a-chip” technology, which represents a new avenue in the field of advanced *in vitro* models, with the potential to revolutionize current approaches to drug screening and toxicology studies. This review aims to highlight recent advances of microfluidic-based devices towards a body-on-a-chip concept, exploring their technology and broad applications in the biomedical field.

## 1. Introduction

Twenty years after the first definition of tissue engineering (TE) came out [1], tissue engineers are now facing new challenges concerning the standardization of the production protocols, cost reduction and up-scaling of these standardized procedures to the clinical setting. Most remarkably, knowledge deriving from tissue engineering is finding increasing application in the development of micro-engineered models of human tissues and organs, which are being investigated as potential alternatives to animal models in elucidating the biological mechanisms underlying morphogenetic and pathogenetic processes, as well as drug screening platforms [2,3,4]. In this scenario, tissue engineering adds the third dimension (3D) to *in vitro* cell cultures, better mimicking the complexity of native tissues and giving access to full-human models. 

Major advances in this field are related to the integration of tissue engineering with microelectronics, microfabrication and microfluidics. Electronic devices have been employed as integrative systems for tissue engineering research. Biosensors, initially dedicated to the detection of biomolecules such as proteins [5,6], peptides [7,8], enzymes [9,10] and DNA [11,12], are now proposed in the tissue engineering field as tools to monitor cell behavior on a miniaturized scale, with high sensitivity and resolution and low associated costs [13,14,15]. By detecting cellular analytes, electrical activity, physical and chemical signals transmitted by the cells, biosensors can provide insights into cellular activities and responses in real time. Therefore microfluidic-based biosensors—also known as lab-on-a-chip (LOC) and Biological/Biomedical Micro Electro Mechanical Systems (BioMEMS)—are becoming more and more popular.

Microfluidic-based biosensors consist of devices in which the manipulation and analysis of fluids occur within micrometer-sized channels [16,17]. Thanks to this miniaturization, the applications of microfluidic devices are countless. To date, microfluidics has been successfully in monitoring and controlling diagnostics [18], cell manipulation [19,20] and drug delivery [21]. Furthermore, the most advanced microfluidic devices not only allow for the monitoring of signals but also actively respond and adapt to them. Here, we highlight and summarize current cutting-edge research on microfluidic devices, their application at a 3D level in tissue engineering and recent developments towards body-on-a-chip concept.

## 2. Microfluidics—from Small Benchtop Biosensors to High-Throughput Systems

Although the concept of *microfluidics* is associated with a framework of complexity and robustness, its roots date back to the 1950s, principally for what concerns inkjet printing technology. As the name suggests, microfluidics is the science and technology associated to the control and manipulation of liquids at a scale of few microliters. Due to the associated advantages of reduced sample volume, scalability, laminar flow and hence highly predictable fluid dynamics, high resolution and sensitivity, short time of analysis, and low cost, there are innumerous fields where microfluidics can be useful and are actually applied. 

In addition to faster medical diagnostics [22,23,24,25], microfluidics is being applied in drugs of abuse testing [26,27,28,29], pollutant detection [30,31,32,33,34], combatting biowarfare [35,36,37,38] and also in laboratory routines in a research context, as described below.

Microfluidics is bound to fit the needs of researchers mainly due to its high-throughput capacity to scale up the number of assays in an automated manner and integration capacity in large experimental pipelines, while reducing the costs. A good example of how this compromise is kept is given by the microfluidic chromatographic column developed by Shapiro and collaborators to test several separation conditions for biopharmaceuticals [39]. 

On the other hand, with the aim of reducing the experimental costs and reagent volumes while keeping the high-throughput capacity of the system, Chen and Ismagilov have developed an alternative to 96-well plates for drug screening, using microfluidic cartridges pre-loaded with nanoliter plugs of reagents [40]. This technology could be applied to biological and chemical assays with associated low cost and simplicity. 

In addition, assays that require thermocycling could also be sped up by using microfluidics technology, as reviewed by Zhang and Xing [41]. Short-term assays, low reagent consumption and rapid heating/cooling rates are some of the advantages of miniaturized PCR devices, which are assets in applications like the molecular diagnostics of diseases [42,43,44] and gene expression analysis [45,46,47,48].

Some of the drawbacks found in conventional research are related with sample manipulation, destabilization of measured signals due to interventions to load a sample or to change a buffer, coupled to the time-consuming experiments and mostly due to the lack of processes automation. By integrating and automating standard laboratory routines, microfluidics technology allows to overcome these limitations, saving time, resources and improving the quality and reproducibility of the results. Indeed, instrument and protocol up-grade to integrate microfluidic platforms allows incorporating several experimental setups into a simplest one with synchronized assay execution and data record. Due to its great impact in research improvement and efficiency, this technology has been adopted by several research groups, increasing the degree of complexity of the assays but also yielding more reliable and reproducible results. 

For instance, Mellors and collaborators developed a fully integrated microfluidic platform to perform high efficiency capillary electrophoresis and electrospray ionization mass spectrometry analysis, useful for proteomics applications [49]. On the other hand, Focke *et al.* described microfluidic cartridges for DNA purification and genotyping, by using standard laboratory instruments as integrative systems [50].

New microfluidic platforms appear every day as a toolbox for the development of new solutions, either to solve benchwork issues or to meet biomedical needs. The complexity and characteristics of the systems obviously depend on the application-specific requirements, varying from very simple devices to engineered complex platforms. 

Paper-based microfluidics systems are the simplest technology for point-of-care diagnostics, combining the well-known methods of lateral flow tests with paper microfluidic technology, where a thin sheet of porous material is the substrate for the bioassays, taking advantage of the substrate high internal surface area, capillary action and absorptive capacity [51]. Notably, the dramatic reduction of costs brought in by paper-based microfluidics holds promise to bring point-of-care diagnostics to developing countries [52].

Microfluidics finds an application in the standardization of cell culture protocols and in the setup of reliable and sensitive bio-sensing assay protocols. Indeed, cells need optimal physiological conditions (pH, temperature and CO_2_) to ensure their viability and activity, they must be continuously and uniformly perfused with nutrients and oxygen and precautions are needed to avoid biofouling effects [53] and side reactions due to non-specific adsorption of biomolecules [54]. Microscale fluid regulators as valves, pumps, mixers and other functional elements allow cell perfusion with fresh media and assay reagents. Furthermore, automated liquid handling, electronic control of switches and valves, multiplexing capability and appropriate detectors to monitor cellular stimuli make a high-throughput screening format feasible. The wide range of different laboratory activities, which already benefit from advanced systems in microfluidics-dependent cell assays, is reviewed elsewhere [55,56].

## 3. Convergence between Microfluidics and Tissue Engineering: Bio-MEMS and Organ-on-a-Chip

BioMEMS are increasingly contributing to TE by providing accurate control of the cell environment in settings suitable for cell screening and by enabling the engineering and studying of minimally functional modules of complex tissues [57]. Although definitions are somewhat overlapping, this last approach is also commonly defined as “organ-on-a-chip” (OoC).

**Figure 1:**
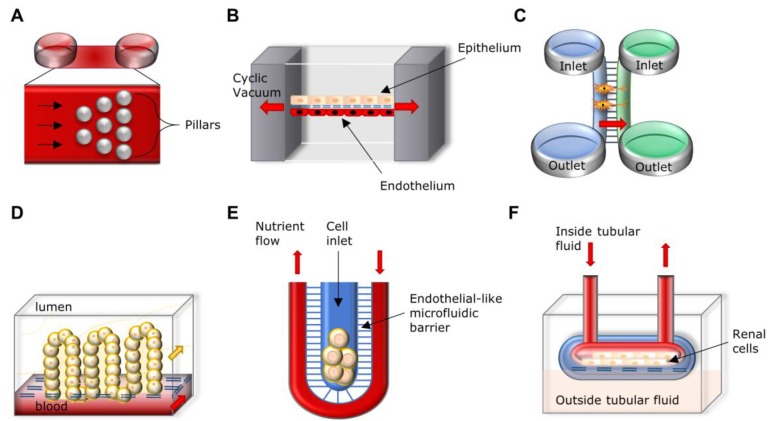
Illustration of the diverse microfluidic devices used to study biological processes occurring in vascular, respiratory, nervous, digestive and excretory systems. **A.** Biochip with subdividing interconnecting microchannels (array of pillars) that decrease in size to mimic cell flow and adhesion in microvasculature to study of vaso-occlusive processes. **B.** Human breathing lung-on-a-chip microdevice, a biomimetic microsystem that reconstitutes the alveolar-capillary interface of the lungs. The device uses compartmentalized chambers to form an alveolar-capillary barrier on a porous membrane and produces cyclic stretching of such membrane by vacuum actuation. **C.** Two-compartment microfluidic culture system bridged by microchannels. It allows the visualization of cell interactions in co-culture, namely as a model for synaptic connectivity between mixed hippocampal co-cultures in which microgrooves allow both axons and dendrites to enter and form synapses. **D**. Vertical cross-section representing the on-chip generation of intestinal villi obtained by villus morphogenesis of Caco-2 cells. The up-scale of this system leads to the production of gut-on-a-chip platforms to study pharmacokinetics and diffusion processes. **E**. Artificial liver sinusoid with a microfluidic endothelial-like barrier for primary hepatocyte culture to study diffusive nutrient transport in liver-mediated metabolism. This unit consists of a cord of hepatocytes fed by diffusion of nutrients across the narrow microfluidic channels from a convective transport vessel. **F.** Kidney proximal tubule-on-a-chip. The microfluidic device consists of an apical channel separated from a bottom reservoir by a porous membrane upon which primary human proximal tubule epithelial cells are cultured in the presence of apical fluid shear stress. This design mimics the dynamically active mechanical microenvironment of the living kidney proximal tubule and allows the study of active and passive epithelial transport.

In this chapter, we highlight the features of BioMEMs as *in vitro* models of cardiovascular, respiratory, nervous, digestive, endocrine and integumentary systems and pathologies (Table 1, Figure 1). 

**Table 1:** Recent applications of BioMEMs.

Application	Platform	References
**Cardiovascular System**		
**Angiogenesis studies**	Dual channel chip/angiogenesis model, microfluidic tri-culture platform	[58,59]
**Biophysical studies**	Pressure attenuator + Funnel chain/cell deformability microfluidic device	[60]
	Muscular thin films	[61]
	Microfluidics + optical microscopy	[62]
	Microfluidics + ultrasound imaging system	[63]
	High-speed video microscopy in microcapillaries	[64]
**Drug screening/development**	Microchannel microfluidic chip	[65]
	Laminar ventricular muscle-on-a-chip	[66]
**Organ/tissue structure/activity**	Microfluidic cardiac cell culture model, heart-on-a-chip, artery-on-a-chip, microscale blood vessel module (µBVM) in a single microchannel device, microfluidic perfusion cell culture chip, microfluidic delivery system, microchannel biochips as vaso-occlusive processes model, perfusion microfluidic device, branched microfluidic channels	[61,67,68,69,70,71,72,73,74]
**Respiratory system**		
**Biological barriers**	Flow stretch chip	[75]
	Compartmentalized microwells in a microfluidic device	[76]
**Cancer mechanisms**	Microfluidics + electric fields	[77]
**Cell culture**	Biomimetic microfluidic airway model	[78]
**Cell differentiation**	3D gelatin-microbbuble scaffold produced by microfluidic device	[79]
**Cell migration**	Dynamic transwell microfluidic system + perfusion culture, microfluidic gradient generator	[80,81]
**Drug delivery**	Microfludics + surface acoustic wave (SAW) nebulizer	[82]
***In vivo* organ studies**	Microfludics + single oxygenator units	[83]
**Molecular mechanisms**	Microfluidics + concentration gradient generator	[84]
**Wound healing**	Microfluidic system of converging multichannels + hydrodynamic flow focusing	[85]
**Nervous System**		
**Axonal transport**	Microchannels/microgrooves + compartmented microfluidic culture	[86,87,88,89]
**Cell culture**	Microchannels/microgrooves + compartmented microfluidic co-cultures, shear-free microfluidic gradient generator	[90,91]
**Cell line characterization**	Microfluidics + electrophoresis	[92]
	Microfluidics + quantitative reverse transcriptase polymerase chain reaction (qRT-PCR)	[93]
**Cell differentiation**	Microgrooves + neuronal compartment + myelination compartment microfluidic co-cultures	[94]
**Cell migration**	Microfluidic microgrooves + compartment to culture explants + compartment with Matrigel^®^ to receive migrating neurons	[95]
**Cellular/Molecular mechanisms**	Two-compartment microfluidic culture system (neuronal compartment + myelination compartment) microfluidic co-cultures, microfluidic axon-microglia platform, axon injury micro-compression platform	[94,96,97,98]
	Microfluidic devices or bioreactors + ultra-performance liquid chromatography-ion mobility-mass (UPLC-IM-MS)	[99]
**Drug delivery**	Microfluidic + perfusion device	[100]
**Drug screening/development**	Microfluidic “Fish-Trap” array, gravity-induced flow + microfluidic chip	[101,102]
	Microfluidics + trans-endothelial electrical resistance (TEER)	[103]
**Organ/tissue structure/activity**	Microfluidic “Fish-Trap” array, two-compartment + microchannels microfluidic culture system	[90,101]
**Screening / Diagnostic**	Microfluidic cell sorter	[104]
**Synaptic studies**	Three compartment microfluidic device competition experiment, two cell culture chambers + funnel-shaped micro-channels microfluidic device	[105,106]
**Toxicity studies**	Axonal microfluidic chambers	[107]
	Microfluidics + 96-well plate	[108]
**Digestive + Excretory System**		
**Cell culture**	Biomimic hydrogel nephron	[109]
	Integrated Dynamic Cell Culture Microchip (IDCCM), Microfluidic endothelial-like barrier, dam-wall and nozzle microfluidic device, hemi-coaxial-flow channel microfluidic, dual perifusion platform	[110,111,112,113,114]
	Microfluidic bioreactor	[115,116,117]
	Microfluidic droplet-based cell encapsulation	[118]
	Multiwell culture system	[119,120]
	Microfluidic-multilayer device (MMD)	[121]
**Cell differentiation**	Microfluidic cell culture chamber/channels	[122,123]
	Microfluidics + qRT-PCR	[124]
**Circulating tumor cells studies**	Microfluidic geometrically enhanced mixing chip, Geometrically Enhanced Differential Immunocapture (GEDI) device	[125,126,127]
**Drug screening/development**	Gut-on-a-chip, 3D villi scaffold + microfluidic device, IDCCM	[128,129,130,131]
	Microfluidics + optical fiber	[132]
	Microfluidic cell culture array	[133]
	Microfluidic droplet-based cell encapsulation	[118]
	Three-dimensional microfluidic microanalytical micro-organ device (3MD)	[134,135]
**Food analysis**	Microfluidics + Fluorescence imaging	[136]
**Metabolism studies**	IDCCM, two-plate bioreactor, metabolomics-on-a-chip, microfluidic delivery device, two-color detection microfluidic system, multimodal islet hypoxia device	[110,117,131,137,138,139,140]
	Microfluidic bioreactor	[141]
	Microscale cell culture analogue (μCCA)	[142]
	Microfluidics-optical sensor	[143]
	Multiwell culture system	[119]
**Organ-organ interaction**	Integrated Insert in a Dynamic Microfluidic Platform (IIDMP), on-chip small intestine-liver coupled microfluidic network	[144,145]
**Screening/Diagnostic**	Microfluidics + surface plasmon resonance	[146]
	Microfluidics + optoelectronic sensor	[147]
	Microfluidics + optomechanical metric	[148]
**Therapeutic systems**	Wearable ultrafiltration units for dialysis	[149,150]
**Toxicity studies**	Metabolomics-on-a-chip, Gut-on-a-chip, IDDCM bioreactor, pharmacokinetic microfluidic perfusion system	[137,151,152,153,154]
	Kidney and kidney/liver microfluidic biochips	[155,156,157]
	Microfluidics + optical fiber	[132]
	μCCA	[142,158]
	Microfluidic bioreactor	[159]
	Microfluidic human kidney proximal tubule-on-a-chip device	[160]
	MMD	[121]
	Multiwell culture system	[119]
**Endocrine System**		
**Cancer mechanisms**	Microfluidic co-culture model, chemokine gradient + 3D culture device	[161,162]
**Fertilization**	Motile spermatozoa sorter + microfluidic chip, microfluidic device mimicking female reproductive tract	[163,164]
**Metabolism studies**	Microfluidics + resonant waveguide grating (RWG) sensor	[165]
**Monitoring**	Microfluidics + electrochemical sensor	[166]
**Screening and diagnostic**	Blood plasma separation microfluidic chip	[18]
	Microfluidics + optical sensor	[167]
	Microfluidics + liquid chromatography-mass spectrometry	[168,169]
	Microfluidics + potentiostat	[170]
	Microfluidics + electrochemical sensor	[171]
	Digital microfluidics	[172]
**Integumentary System**		
**Biological barriers**	Stable gel/liquid interface microfluidic chip	[173]
**Cell differentiation**	Pillar array microfluidic device based on cell surface markers	[174]
**Cell migration**	3D matrices microfluidic device	[175]
**Screening and diagnostic**	Microfluidics + conductometric sensor	[176]
	Microfluidics + potentiometric sensor	[177]
**Skin repair**	Microfluidic wound-healing model + wound dressing screening	[178,179]

In general, such devices are obtained by soft-lithographic processes, with polydimethylsiloxane (PDMS) and glass representing common materials for the fabrication of microfluidic channels, which makes such devices compatible with live-cell microscopy and high throughput screening methodologies.

Devices may also endow porous membranes to compartmentalize different cell populations and biomimetic coatings with extracellular matrix (ECM) components such as fibronectin, collagen, or Matrigel^®^ to improve cell attachment. A comprehensive review of biomaterial-related issues for the fabrication of BioMEMs has been provided by Berthier *et al.* [180].

### 3.1. Cardiovascular System

More than in the direct treatment of cardiovascular pathologies, microfluidics strategies and/or devices are being applied in *in vitro* models, diagnostics, clinical studies and drug screening with the aim of reducing the intervention time and to set up more efficient therapies. Thanks to their conduit-like design, and to their precise control over flow conditions, including shear stress and pulsatility, microfluidic devices are particularly likely to be used as reductionist models of cardiovascular biology (e.g., to mimic blood flow and predict injuries to blood vessels), than to study heart-related issues. Nevertheless, modern biomedical engineering is advanced enough as to reproduce cardiovascular system complexity. Microfluidic cardiac cell cultures are physiologically relevant *in vitro* models that recreate mechanical loading conditions seen in both normal and pathological conditions and allow hemodynamic stimulation of cardiomyocytes by directly coupling cell structure and function with fluid-induced loading [61,67]. In this context, an example of “heart-on-a-chip” was given by using poly(N-isopropylacrylamide) (PIPAAm) and PDMS to engineer an anisotropic rat ventricular tissue and to measure contractility, action potential propagation, epinephrine dose-response and cytoskeletal architecture in a mid- to high-throughput system allowing real time data collection [61].

Exploiting a similar PIPAAm/PDMS-based system, the same research group provided evidence that it is indeed possible to reproduce on chip the negative remodeling of the failing myocardium by applying cyclic mechanical stretch to mimic pathological mechanical overload [66].

Furthermore, if combined with cells or biopsies harvested from patients, these models could be used as tools for drug screening in individualized medicine. The major concern with the setup of microfluidics systems for cardiovascular testing is in the peculiar growth attitude of the cardiomyocytes, requiring special conditions to adhere and survive while preserving their unique contractile phenotype. Thus, the discussion is still open to find the most appropriate and representative source of contractile cells. 

As soon as the vascular component of the cardiovascular system is taken into account, reproducing the complexity of the system itself becomes more and more challenging. Several research groups are interested in the development of microfluidic devices in which angiogenesis [58,59], artery structure [68] and network [69], vascular endothelial function [70] growth and remodeling [71] can be studied. More directed to vascular pathologies, other groups are focused in highlighting vaso‑occlusive processes [72] (Figure 1A) and thrombosis [73], evaluating hypertensive micro vessels [74] and antihypertensive drug effects [65], or studying long-term vascular contractility [71]. 

Numerous blood pathologies are caused by the decrease of red blood cell deformability impeding the transit of these cells through the microvasculature, where they play a central role in the oxygenation of tissues. Therefore, a common indicator of hemorheological dysfunction is the measure of red blood cell deformability or dynamic analysis of blood flow. Biophysical properties including red blood cells aggregation, deformability, viscosity, velocity profile and pressure of blood flows have been measured in systems engineered by Yeom [62,63], Guo [60], Tomaiuolo [64] and Zheng [181,182] and were useful for understanding the effects of hemorheological features on the hemodynamic characteristics in capillary blood vessels.

### 3.2. Respiratory System

Most frequent respiratory diseases act by affecting the airways, the structure of the lung tissue, blood circulation in the lungs, or involve a combination of these three. Given the precise control over fluidic parameters, and the successful modeling of tissue interfaces, microfluidic platforms are finding increasing application in the study of respiratory system pathophysiology.

Some of the first studies have reported biomimetic microsystems reproducing the alveolar-capillary interface of the human lung as an alternative to animal and clinical studies, for drug screening and toxicology applications [75,183,184] (Figure 1B). Since then, several authors have developed biomimetic models, BioMEMs or microfluidic-based devices with the purpose of highlighting and modeling important issues in lung development, differentiation, homeostasis and disease [185]. Recently, two approaches used microfluidic devices to study the differentiation of lung stem/progenitor cells in the view of future lung tissue engineering applications [79,186]. In the first approach, alveoli-like structures were obtained after seeding isolated mouse pulmonary stem/progenitor cells in a compatible gelatin/microbubble-scaffold using a 2-channel fluid jacket microfluidic device [79]. The second strategy consisted in the development of microfluidic magnetic activated cell sorting system in the isolation of mouse lung multipotent stem cells for further characterization [186]. Different research groups are focused on the development of models that mimic lung barrier and, in combination with cells from patients, are proposed as drug-screening platforms to select candidate drugs to treat pulmonary pathologies [76,78].

As far as the onset of lung diseases is concerned, researchers are focused in producing biomimetic microsystems so that the molecular processes underlying pathologies such as malignant transformation of bronchial epithelial cells due to tobacco [84], protein-induced lung inflammation [80], chronic obstructive pulmonary disease [81] and idiopathic pulmonary fibrosis [85] can be highlighted. 

Applications of microfluidics also regard the development of implantable respiratory assist devices with a potential for clinical application. As an example, in the last few years, Kniazeva and Hoganson described a small-scale microfluidic artificial lung and an implantable ambulatory lung assist device based on stacked microchannel networks, ultrathin gas exchange membranes, and with the potential to be used in the clinics [187,188,189,190].

While microfluidic artificial lung is still under development, several miniaturized devices are now closer to being translated to the clinical application. Cortez *et al.* developed a portable acoustomicrofluidic device capable of nebulizing drugs into a fine aerosol for deep lung deposition via inhalation with negligible drug degradation, as successfully demonstrated in the case of epidermal growth factor receptor (EGFR) monoclonal antibodies [82]. Also, Rochow engineered a miniaturized oxygenator device, composed of stacked single microfluidic units and perfused like an artificial placenta via the umbilical vessels, that might support newborns with respiratory insufficiency [83].

### 3.3. Nervous System

Nervous system pathologies have their origin in aging, genetic alterations, brain trauma and spinal cord injuries, among others. Given the intrinsic complexity of the nervous system, one of the most described applications of microfluidic technology is represented by *in vitro* models mimicking the nervous tissue-vasculature interaction. Microfluidic platforms have been described as ideal *in vitro* cerebrovascular models not only due to their automatized features, miniaturized scale and low cost, but also because of their ability to mimic physiological dynamics, physical properties and biological microenvironment complexity [191]. Generally, such devices can be applied to model and study the progression of neurodegenerative diseases and screen drug candidates towards individualized medicine solutions. 

In particular, the impairment of blood-brain barrier (BBB) is considered to be among the main causes of pathogenesis and/or progression of several neurological disorders such as epilepsy, multiple sclerosis, Parkinson and Alzheimer’s disease. Therefore, a better understanding of the physiology, microenvironment, cell-cell interactions at the BBB level can provide important clues on brain disorders or help designing and testing efficient drug candidates. In 2012 Booth upgraded the static (transwell) *in vitro* model of BBB to a dynamic *one*, and then used it to analyze neuroactive drugs [103,192], establishing a versatile model for prediction of BBB clearance of pharmaceuticals. 

In normal physiologic conditions, microfluidics-based *in vitro* models can contribute to a better understanding of mechanisms behind the formation and function of neuronal networks. Thus, these models allow for the reproduction of synaptic competition [105], cell line authentication [92,93], study of neuronal migration in embryonic brain explants [95], axonal guidance during brain development [93] (Figure 1C) and myelination [94]. The use of brain explants within microfluidic devices also allows for the exposure to multiple compounds at once or in sequence, thus improving the existent models towards an individual medicine approach as a guided therapeutic decision-making [100,104,108]. Of great interest is the possibility to exploit microfluidics for high-throughput mapping of brain-wide activity in awake and drug-responsive vertebrates (e.g., zebrafish) [101].

To visualize the fundamental physiological changes occurring during the onset of neurodegenerative diseases, microfluidic systems were developed to model synaptic connectivity between mixed hippocampal co-cultures [90] (Figure 1C), to reconstruct neuronal network and test β-amyloid toxicity [102,106] as well as to follow the activation of developmental brain disorders [99]. At the axonal level, structural and functional deficits are predictive of an early occurrence of neurodegenerative diseases. Therefore several platforms have been designed to highlight mechanisms of axonal function impairment [97], axon-polarization [91], axon toxicity [107], deformation [98], and to trace axonal transport at single vesicle level [86,87,88,89].

### 3.4. Digestive and Excretory Systems

A variety of diseases negatively affecting digestive system lead to gastrointestinal organ damage and function deterioration. Stomach and esophagus cancer, short bowel syndrome, fecal incontinence and trauma are among the pathologies affecting gastrointestinal function and urging for a treatment. In the early diagnosis context, Zilberman and Sonkusale stood out with a strategy based on optoelectronic sensors for early gastric cancer detection in saliva, thus proposing an alternative non-invasive method to endoscopy, biopsy and histopathological evaluation [147].

Since conventional 2D culture systems lack reproducibility of chemical complexity and biofunctionality of the living tissues, microfluidics arose as an alternative platform to develop strategies to settle gastrointestinal tissue regeneration and study organ physiological functionality. Due to the structure and dynamic features of BioMEMs, there is a great interest in the use of these systems to more accurately study the intestinal absorption of drugs and their toxicity. For instance, Kimura and colleagues developed an integrated microfluidic system endowing on-chip pumping and optical fiber detection systems. Performance of the device was examined through long-term culture and monitoring of polarized transport activity of Caco-2 cells [132]. Mahler *et al.* [158], as well as McAuliffe and collaborators [142], have also contributed to the design of drug transport models using microfluidic devices.

Microfluidic complex systems to create *in vitro* models of the intestine are valuable tools to study gut function under normal or diseased conditions and also to perform drug screening and toxicity assays. In this regard Kim and co-workers developed a microengineered “human gut-on-a-chip”, a system composed by two microfluidic channels with a flexible porous membrane coated with extracellular matrix, lined by gut epithelial cells (Caco-2), making it possible to recreate the gut structure with its mechanical, absorptive, transport and pathophysiological properties [151]. One year later, Kim and Ingber demonstrated that applying specific physiological mechanical cues to the gut-on-a-chip, it was possible to induce Caco-2 cells to spontaneously undergo intestinal villi morphogenesis [128] (Figure 1D). This model was recently upscaled by the development of a platform that can be adaptable to produce several functional units of other organs [152]. Further improvements in the recapitulation of the “intestinal epithelium-on-a-chip” consist in the fabrication of 3D-shaped microporous polymeric membranes mimicking the geometry of the intestinal villi [129] or by the so called “intestinal epithelium-on-a-chip” being reproduced by using a novel hydrogel microfabrication technique and showing a superior structural maturation [130]. Their microfluidic device was further used to study the kinetics of diffusion processes in the 3D villi scaffold. A much higher degree of complexity was reached by Ramadan and collaborators with the microfluidic platform called *NutriChip*. With the aim to analyze the passage of nutrients through the gastrointestinal tract (GIT), they developed a miniaturized GIT including the epithelial and immune cell components, in which the response of immune cells to pro and anti-inflammatory stimuli was monitored [130].

The liver also plays an important role in the digestion processes, being responsible for the filtration of nutrients and digestion products. Furthermore, liver represents a fundamental key in the metabolism of xenobiotics and thus a number of publications came out in the last years, describing strategies to recreate liver-specific functions through microengineered models, with the purpose of studying drug metabolism and ultimately improving drug development processes [118,119,120,134,135]. Moreover, numerous publications described the use of microfluidics for the development of physiologically-relevant hepatocyte cell culture [110,111,115,117] (Figure 1E), differentiation [122] and co-culture systems [117,132,144,193] as well as the design of platforms for diagnostic applications [146]. Additionally, the development of microfluidic-based devices to investigate liver drug metabolism and toxicity [131,133,137,153,154,159,194] are to be considered fundamental tools to address liver pathologies, better understand molecular toxicity mechanisms and simulate drug-drug and organ-organ interactions. A comprehensive review about this topic is given by van Midwoud and colleagues [195].

Pancreas also plays an important role in the digestion process, as it is responsible for producing enzymes and hormones to be secreted into the small intestine. Deficient production of digestive enzymes and hormones, as well pancreas blockage by tumors and gallstones, leads to subsequent malfunction of the entire digestive system and further compliances. Trying to solve these life-threatening conditions, researchers have made use of microfluidics to study and diagnose pancreatic cancer [125,126,127,148], culture pancreatic islets [112,113,114,138], monitor stimulus-secretion factors [139,140,143] and promote tissue-specific cell differentiation [124]. 

The perfect example of how microfluidics can be successfully applied to treat pancreatic dysfunctions comes from the “bionic pancreas” developed for type 1 diabetes, that uses continuous glucose monitoring along with subcutaneous delivery of both rapid-acting insulin and glucagon to lower/increase blood glucose levels [196].

Due to the important functions on processing digestion products, water balance and blood pressure regulation, kidneys are of fundamental importance for whole-body homeostasis. Therefore, there is a considerable interest to develop strategies to adequately treat the most problematic conditions affecting kidneys. Among them, chronic kidney disease often results in end-stage renal failure, requiring renal replacement therapy and eventually transplantation, causing a massive burden on the healthcare systems. Microfluidic systems, as the one developed by Leonard and collaborators, appear as innovative tools to improve the outcome of classical approaches [150,151]. For instance, a membraneless dialysis strategy was developed, opening possibilities to create wearable blood processing devices [150,151]. Other microfluidic systems enable the culture of kidney cells in tubular structures, mimicking the organ structure and function [109,121,123,160] (Figure 1F). The potential of application of microfluidic also includes disease modeling and metabolism studies, giving insights about kidney cell toxicity and renal clearance [141,155,156,160]. Renal excretion and metabolism are the actual subjects of preclinical safety studies, with the goal of investigating drug pharmacokinetics in *in vivo*-like pathophysiological conditions. Therefore, microfluidic devices can be useful to co-culture different cell types [157] with particular impact on the recreation of multi-organ systems to study systemic interaction where kidneys and also liver can be incorporated. A wide perspective on multi-organs-on-a-chip is described in Section 4.

### 3.5. Other Promising Applications for Microfluidics Technology

Although being still in their very early developmental stage, some microfluidic platforms appear as innovative systems for significant endocrine studies. Concerning the adrenal glands, microfluidics is being applied to detect and study corticosteroids [167,168,170] and catecholamines [165,166,171]. In the fertility context, Huang and collaborators used microfluidics to isolate, analyze and quantify spermatozoids [163]. By a similar approach, Tung and collaborators demonstrated that the biophysical environment of female reproductive tract critically guide sperm migration without aiding the migration of pathogens [164]. Kim, Broccardo and co-workers used microfluidics to quantify steroid hormone levels in tissue [169] and in human serum [168], what can be relevant in fertility and osteoporosis studies. Microfluidic systems have been also employed with the aim to diagnose thyroid diseases, as described by Shamsi and co-workers as well as by Madadi and colleagues [18,172]. Further studies employing microfluidics platforms were performed in hormonally-responsive cancers. Lang and colleagues explored breast cancer microenvironment activity using protein levels as a sensor to predict how cell signaling is related with the growth of cancer cells [161]. On the other hand, Kim and co-workers examined how chemoinvasion processes are affected by chemical gradients, studying tumor cell migration behavior to understand the first steps of cancer metastasis [162].

Microfluidics also appears as an innovative application in the wearable sensors for continuous physiological signals monitoring. Sweat, as a non-invasive biofluid, is the subject of intense investigations in this context. For instance, Rose and collaborators, as well as Liu and colleagues, developed sensor patches for sweat electrolytes monitoring and aiming at hydration control [176,177]. In turn, Xu and co-workers described experimental and theoretical approaches for soft microfluidics assemblies in sensors, circuits and radios for the skin [197]. Furthermore, the group of Sonner has recently reviewed microfluidics models for eccrine sweat generation and flow, as a guide for sweat-based diagnostics development [198]. Other recent approaches to investigate the function and deficits of integumentary system comprise the microfluidics platform developed to study the accumulation of molecules at the basal lamina interfaces and achieve efficient drugs and carriers’ distribution through biological barriers [173]. Microfluidics applications to model skin diseases and for skin tissue regeneration are still in an early stage. However, some works in wound healing [174,178,179] and cell migration [175] showed that this technology may have potential to treat skin injuries.

## 4. Body-on-a-Chip: A Future Perspective

**Figure 2:**
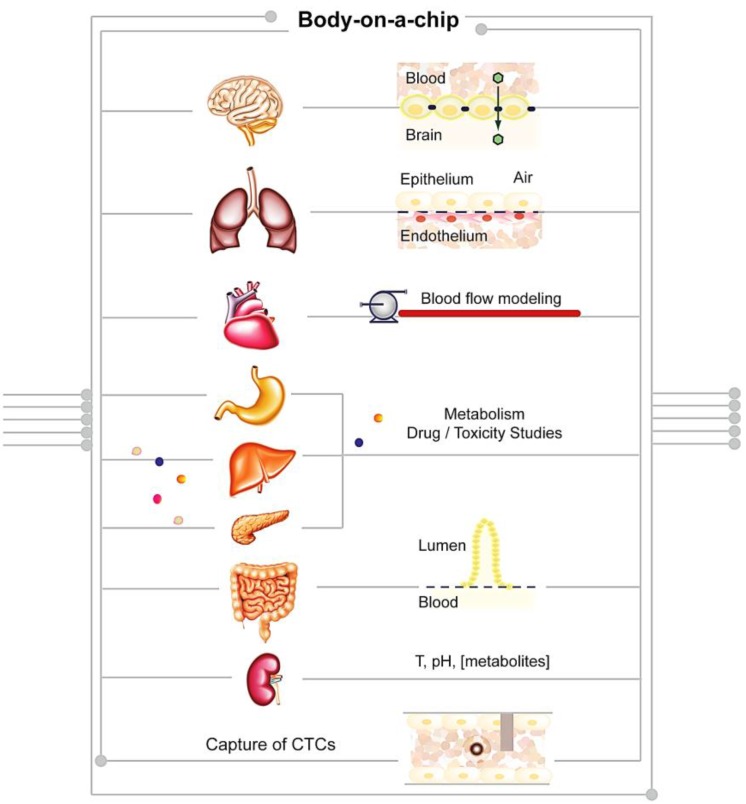
Schematic representation of a BoC approach in which cell-autonomous and non-autonomous studies can be performed using a single chip.

According to a recent analysis by Scannell *et al.*, the past 60 years have seen huge advances in many of the scientific, technological and managerial factors that should tend to raise the efficiency of commercial drug R&D [199]. Yet the number of new drugs approved per billion US dollars spent on R&D has halved roughly every nine years since 1950, falling around 80-fold in inflation-adjusted terms. Improving the effectiveness of preclinical predictions of human drug responses is critical to reducing costly failures in clinical trials. As evidenced in the previous section, recent advances in tissue engineering, microfabrication and microfluidics have enabled the development of microengineered models of the functional units of human organs. This approach is believed to provide the basis for preclinical assays with greater predictive power [3]. This concept can be further extended, recapitulating the function of several organs on a single microfluidic platform, with the final goal to mimic the whole body physiology. Therefore, the Body-on-a-Chip (BoC) concept is gaining relevance as a suitable device to study and predict cell-drug and cell-cell response [200]. BoC devices consist of microfluidic chips into which several modules can be installed holding different cell types or engineered human organs [201]. Samples are interconnected in a hierarchic and physiologically relevant fashion, thus allowing the functional modeling and monitoring of the circulatory, endocrine, digestive, immune, lymphatic, nervous, respiratory and urinary systems, as an advanced human *in vitro* model (Figure 2).

Since BoC models mimic physiological context and key aspects of human metabolism, they allow for:

- high accuracy prediction and comprehensive analyses of novel therapeutic candidates during preclinical stages, by a closer estimation of efficacy and dose response;
- reduction and likely replacement of animals in preclinical drug development, thereby reducing costs and time to market;
- creation of a drug development tool that helps modern medicine rapidly respond to fast-moving pandemics or chemical warfare/bioterrorism attacks;
- study cell signaling by monitoring the metabolites that are consumed, produced, and exchanged between all tissues at physiologically relevant concentrations in real time;
- study embryology and its signaling pathways by following intercellular signals and/or bioelectrical messages;
- conduct experiments that cannot be performed in cell culture, e.g., study of tissue-tissue interactions that occur as a result of metabolite travelling from one tissue to other distant tissue, and through dynamic forces that resemble blood circulation;
- efficient and reliable cell–cell and cell-drug/biomaterial interaction studies, narrowing the gap between *in vivo* and *in vitro* conditions.

Microfluidics can bring more benefits if complemented with sensitive analytical methods (namely mass spectroscopy and sensors), enabling the metabolic profiling and comprehensive molecular characterization of the chip-based cell systems. Furthermore, as the chip channels are usually transparent, it is also possible to monitor cell response and perform cell-tracking through time lapse live-cell imaging.

It is important to recognize that there are two complementary approaches for BoC development. Bottom-up approaches start from a detailed specification of each organ, and then proceed with the design of coupled systems (e.g., heart–lung and intestine–liver), adding organs to create more complex models. Top–down approaches, on the contrary, consider the abstract, system-level architecture of an organism and then break the system down into the functionality of compositional organ systems. 

Moreover, it is also possible to explore the process of inflammation response by adding cytokines or living immune cells to the system [202,203]. Also, BoC devices with biopsy samples or cells from individual patients can be very helpful in the development of individualized medicine to predict how the patient might react to a certain pharmacological treatment, prior to administration, thus reducing risks [204]. 

### 4.1. Proposed Applications of BoCs

As listed in Table 2, several BoCs are currently being developed. For instance, BoC simulation with gastrointestinal tract and liver tissues was prepared by co-culturing Caco-2 enterocytes, TH29-MTX mucin-producing cell line and HepG2/C3A hepatocytes in a microfluidic device. The results suggested that ingested carboxylated polystyrene nanoparticles have the potential to cause liver injury, thus showing that BoC devices are highly relevant *in vitro* multicellular models for evaluating nanoparticle interactions with human tissues [200]. 

Vunjak-Novakovic and her team developed the *HeLiVa* platform, an integrated heart-liver-vascular system derived from a single line of human pluripotent stem cells and enabling the functional representation of human physiology in combination with real-time biological readouts and compatibility with high-throughput analysis [205]. In the same pharmacological context, the first pass intestinal and liver metabolism of paracetamol in a microfluidic platform coupled with mathematical modeling as a means to evaluate absorption, distribution, metabolism, and excretion (ADME) processes in humans was described by Prot and collaborators [206]. The overall approach provided a first step in an integrated strategy combining *in silico* and *in vitro* methods based on microfluidics for evaluating drug ADME processes [206]. Approximately one year later, a four-organ-chip for interconnected long-term co-culture of human intestine, liver, skin and kidney equivalents was introduced [207]. The system guarantees near to physiological fluid-to-tissue ratios and the establishment of reproducible homeostasis among the co-cultures, sustainable over at least 28 days. This system thus qualifies as a powerful tool to perform *in vitro* microfluidic ADME profiling and repeated dose systemic toxicity testing of drug candidates [208]. Pursuing to design a system with a higher degree of complexity, a 96-well format-based microfluidics platform was prepared to interconnect various multicellular 3D spheroids, which enables parallelized culturing and testing of spherical microtissues of different cell types in a standard incubator [209]. This kind of device allows for the study of tissue-tissue interactions in the presence of pharmacologic components [210,211]. According to the manufacturer, Swiss startup *InSphero^®^*, the commercial multi-tissue device could be ready in three years. 

Exploiting the possibility to connect different chips to increase the physiological relevance of the *in vitro* system, efforts are being made in recreating whole human body by bridging the fluidics of multiple chips, so that the physiological pharmacokinetics of the drugs of interest can be studied in a very complex, representative *in vitro* system.

**Table 2:** Available BoCs and their applications.

Organs / Interactions	Device / Platform Name	Application	References
**Brain, Heart, Lung, Skin, Adipose, Muscle, Liver, Bone Marrow, Kidney**	Physiologically-based pharmacokinetic (PBPK) model	ADME profiling and quantification of the amount of drugs in different parts of the body	[212,213]
**Gastrointestinal Tract and Liver**	µCCA	Evaluating nanoparticle toxicity and interactions with tissues	[200]
	Gut—parallel tube model	Investigate paracetamol intestinal and liver first pass metabolism	[206]
**Heart, Liver, Vascular System**	HeLiVa	Drug testing in human health and disease	[205]
**Intestine, Liver, Skin and Kidney**	Four-Organ-Chip	ADME profiling and toxicity testing	[207]
**Liver, Colorectal Tissues**	96-well format-based microfluidic platform	Testing drug effects at different concentrations in several tissues	[209,210,211]
**Liver, Heart, Lung and Kidney**	ATHENA (“Homo Minutus”)	Screening new drugs for potency and potential side-effects	[214]
**Liver, Tumor and Marrow**	Pharmacokinetic-pharmacodynamic (PK-PD) model combined with a µCCA	Testing drug toxicity and improve insights into the drug’s mechanism of action	[212]
**Lung, Gut**	PDMS-based organs-on-chip	Prediction of clinical responses in humans	[152,215]

In a first approach, this pharmacokinetics–pharmacodynamics platform was tested with three cell lines representing the liver, tumor and bone marrow [212], but can also be extrapolated for more organs to predict mammalian response to drug and chemical exposure [213]. 

With a similar aim, the group of Donald E. Ingber is designing organs-on-chip which replicate key functional units of living organs to reconstitute integrated human organ-level pathophysiology *in vitro* [152,215]. The final purpose will be to combine as many organs as possible to closely mimic a real human body. The development of the *ATHENA* (Advanced Tissue-engineered Human Ectypal Network Analyzer) platform, also known as “*Homo Minutus*”, in which four interconnected human organ constructs (liver, heart, lung and kidney) are interconnected in a highly miniaturized platform follows the same principle [214]. This “Benchtop Human” is a big promise as it has the ability to simulate the spatial and functional complexity of human organs, leading to a more accurate way of screening new drugs for potency and potential side effects than current methods [216].

### 4.2. BoCs and Cancer

The interest in disclosing the signals peculiar of the cancer microenvironment and influencing tumor cell growth, malignancy [217,218,219,220,221] and transvascular migration [222,223], is growing steadily together with the search for new cancer prevention and diagnostics tools [224]. Scientists are aware that tumor cell migration and intravasation into capillaries is an early and key event in cancer metastasis. Therefore, several platforms have been developed with the aim to efficiently detect and harvest circulating tumor cells (CTCs) and clusters, and for chemosensitivity or chemoresistance assays. In fact, several works report the development of microfluidic devices for the isolation of CTCs from lung [221,224,225,226,227], pancreatic [125,126], breast [217,226,228], ovarian [217,229], prostate [217,229,230], colorectal [231], gastric [228], hepatic [232] and skin (melanoma) [229] cancer. 

Concerning lung cancer, it is possible to characterize the early stages of progression while predicting the occurrence of metastasis using CTCs from patients [233]. Also, to provide an inexpensive and effective tool for CTC detection and evaluation of cancer status, Huang and collaborators developed a microfluidic size-based sorting platform, with the advantage of capturing tumor cells without taking into account the expression of specific cell surface markers [234]. 

With a different aim, Ying and collaborators fabricated a 3D microfluidic chip generating a concentration gradient of hepatocyte growth factor (HGF) to investigate its impact on Met/PI3K/AKT activation, glucose regulatory protein expression and paclitaxel-induced A549 cell apoptosis [235], as to mimic the *in vivo* secretion of the growth factor by cancer-associated fibroblasts. Also, in order to modulate chemotaxis and electrotaxis of lung cancer cells, Kao employed direct-current electric fields in a microfluidic cell culture device obtaining both stable electric field and concentration gradients [77]. Recently, trying to be closer to a personalized medicine approach, Ruppen and collaborators demonstrated the possibility to reproduce, at least partly, the barrier induced by the tumor microenvironment to protect the tumor from drug exposure by testing the chemosensitivity of patient lung cancer cell spheroids in a perfused microfluidic platform [236]. Phenomena such as tumor extravasation and metastatic site specificity have also been investigated using 3D microfluidic models [237,238,239].

Several systems allow for the capture of CTCs and clusters from blood samples for further detailed analysis of biomarkers by flow cytometry technology and multi-imaging [205,221,222,223]. Furthermore, the capture of these cells/clusters is suited for the identification of patients with metastatic cancer and RNA sequencing of cancer cells can elucidate about the presence of cell mutations [226] and identify the tumor origin. 

### 4.3. Limitations of BoCs

As thoroughly analyzed by Wikswo *et al.* [240], abstracting the complexity of biology to obtain a meaningful model for studying the properties of the entire system poses significant challenges. Determining the proper size of each organ and its perfusion conditions, vascularizing organ units with proper surface-to-volume ratio, and integrating the system with models describing the states of health and disease, are but a few problems that need to be solved for successful implementation of BoC approach [213,214,240]. 

From a more operational perspective, due to their (micro) scale, BoCs show several limitations, mainly related to the growth of cells in such tiny channels, the formation of air bubbles in the cell culture channels and the hurdles of long-term experiments [152]. Furthermore, the use of 3D organs within the chips is subjected to a high batch-to-batch variability. One other drawback is in BoCs being usually complex systems that require a special skilled operator, so—as to favor their spreading and use on a daily basis—they should be re-designed to be simple, flexible and user-friendly, in order to be employed as benchtop analyzers. In addition, the effects that polymers and fluids utilized in BoCs exert on cell behavior and in the adsorption of metabolites are still poorly understood. That is why one of the challenges scientists will have to address while trying to set up physiological *in vitro* models of diseases by BoCs technology is to establish suitable universal cell culture conditions (a universal ‘blood surrogate’) enabling the preservation of cellular phenotype and function, and providing effective humoral communication between the different cell and tissue types [240]. 

Despite all the barriers to reach commercialization, there are several devices already approved and/or under approval by FDA. For instance, *CellSearch^®^* is considered the first FDA-approved CTC diagnostic technology for clinical use and the only actionable test for detecting CTCs in cancer patients with metastatic breast, prostate or colorectal cancer. This device is already being applied in clinical studies [241]. However, with the high technology advances that are being observed from year to year, the majority of the platforms described in this review may be in a near future optimized to fit FDA rules. 

Comparing the inherent limitations of BoCs with the limitations existent in *in vitro* models and with the complexity found in animal models, BoC devices represent a step forward. Indeed, BoCs play a role as gateways for a comprehensive platform, which allows identifying multi-organ toxicity and/or decreased efficacy due to metabolic activity. BoCs have the ability to not only improve the drug development process significantly, but also to improve the knowledge on tissue-tissue and/or tissue-biomaterials interactions, diminishing the gap between *in vivo* and *in vitro* conditions in tissue engineering applications and disease progression studies.

## 5. Concluding Remarks

The potential of microfluidics to fuel research applications and enter routine clinical practice is indeed impressive. The use of microfluidic platforms as biomedical tools holds the promise to be further implemented as clinically-relevant devices to be included in the daily healthcare practice, by anticipating and monitoring the onset of diseases. Due to the interplay among microfluidics, biosensors and tissue engineering know-how, diagnostics is becoming faster and cheaper, and biomedical devices are getting more comprehensive and able to restore complex lost functions of diseased or damaged tissues and organs.

In research, microfluidics is used as a complement to several methodologies and prototypes to solve bench issues or to improve existing technologies. Several publications describe proof-of-concept devices as innovative and smart alternatives for biomedical applications, although their FDA approval, standardization and further manufacturing in large-scale is still far to come. FDA rules are very restrictive in the direct use of microfluidics for tissue engineering applications; however this technology can be easily adapted to host 3D microtissues and BoC devices, offering better predictability of drug effects than conventional 2D test systems. These models enable a deep understanding of interactions between drugs and their metabolites in various organs with regard to toxic effects and/or drug efficacy. Despite the majority of the biomedical applications of microfluidics being *in vitro* or *ex vivo*, in the near future the use of microfluidic devices will most likely be preferred to *in vivo* studies, and upscaled to be suitable for the diagnostics and clinical scenarios, like in the capture of circulating tumor cells and clusters. 

Microfluidic technology is thus deemed to have a huge impact on science and medicine practice due to its rapid progress, to its tunability and scalability, which leads to outline a trajectory of tremendous innovation with countless potential. From simple to complex systems, microfluidics will be evolving, being part of breakthrough and futurist ideas, thus playing a role in the improvement of organ-on-a chip studies to body-on-a-chip approaches.

## List of Abbreviations

2D—two-dimensional

3D—three-dimensional

3MD—Three-dimensional microfluidic microanalytical micro-organ device

ADME—absorption, distribution, metabolism, and excretion

BBB—blood-brain barrier

BioMEMS—biological/biomedical micro electro mechanical systems

BOC—body-on-a-chip

COPD—chronic obstructive pulmonary disease

CTCs—circulating tumor cells

ECM—extracellular matrix

GEDI—geometrically enhanced differential immunocapture

GIT—gastrointestinal tract

HGF—hepatocyte growth factor

IDCCM—integrated dynamic cell culture microchip

IIDMP—integrated insert in a dynamic microfluidic platform

LOC—lab-on-a-chip

MMD—Microfluidic-multilayer device

OoC—organ-on-a-chip

PDMS—poly(dimethylsiloxide)

PEG—poly(ethylene glycol)

PIPAAm—poly(N-isopropylacrylamide)

PMMA—poly(methyl methacrylate)

PTFE—poly(tetrafluoroethylene)

RWG—resonant waveguide grating

SAW—surface acoustic wave

TE—tissue engineering

UPLC-IM-MS—ultra-performance liquid chromatography-ion mobility-mass

µBVM—microscale blood vessel module

μCCA—microscale cell culture analog
